# L-Serine, an Endogenous Amino Acid, Is a Potential Neuroprotective Agent for Neurological Disease and Injury

**DOI:** 10.3389/fnmol.2021.726665

**Published:** 2021-09-06

**Authors:** Lisha Ye, Yechao Sun, Zhenglin Jiang, Guohua Wang

**Affiliations:** Department of Neurophysiology and Neuropharmacology, Institute of Special Environmental Medicine and Co-innovation Center of Neuroregeneration, Nantong University, Nantong, China

**Keywords:** L-serine, D-serine, neurological disease, neurological injury, neuroprotection, stroke, traumatic brain injury

## Abstract

Central nervous system (CNS) lesions are major causes of human death and disability worldwide, and they cause different extents of motor and sensory dysfunction in patients. Thus, it is crucial to develop new effective neuroprotective drugs and approaches targeted to the heterogeneous nature of CNS injury and disease. L-serine is an indispensable neurotrophic factor and a precursor for neurotransmitters. Although L-serine is a native amino acid supplement, its metabolic products have been shown to be essential not only for cell proliferation but also for neuronal development and specific functions in the brain. Growing evidence has suggested that L-serine regulates the release of several cytokines in the brain under some neuropathological conditions to recover cognitive function, improve cerebral blood flow, inhibit inflammation, promote remyelination and exert other neuroprotective effects on neurological injury. L-serine has also been used to treat epilepsy, schizophrenia, psychosis, and Alzheimer’s Disease as well as other neurological diseases. Furthermore, the dosing of animals with L-serine and human clinical trials investigating the therapeutic effects of L-serine generally support the safety of L-serine. The high significance of this review lies in its emphasis on the therapeutic potential of using L-serine as a general treatment for numerous CNS diseases and injuries. Because L-serine performs a broad spectrum of functions, it may be clinically used as an effective neuroprotective agent.

## Introduction

The central nervous system (CNS) controls the functions of all organs and systems of the human body. When the CNS is damaged, a subsequent cascade of pathological reactions is induced by primary injuries that involve an increase in excitatory amino acids, calcium overload, blood-brain barrier damage, apoptosis, and oxidative stress (Wang et al., 2011; Anthony and Couch, 2014). CNS injury and diseases mainly include traumatic brain injury (TBI), cerebral ischemia and neurodegenerative diseases, such as Parkinson’s disease and Alzheimer’s disease (AD; Gardner and Yaffe, 2015). All of these factors lead to cell death, excessive glial cell activation, demyelination, and axon damage (Xiong et al., 2013; Roth et al., 2014). At the same time, many factors affect the repair of neurological function after CNS injury. For example, inhibitory factors in the extracellular environment include inhibitory ligands released from the myelin sheath and glial cells after injury, as well as glial scars, and they also include the Nogo transmembrane protein and myelin sheath-related proteins. By binding to receptors on the axon membrane, inhibitory ligands interfere with the synthesis of related proteins required by neurons. Moreover, glial scarring also inhibits the regeneration of axons (Cregg et al., 2014; Moeendarbary et al., 2017). All of these factors increase the severity of secondary damage in neurological disease, making it difficult for most patients with a neurological disease to return to a normal physiological state.

The vast majority of CNS diseases also cause extensive neuronal death, but sustained and severe secondary injury can also cause severe damage to white matter, which accounts for approximately half of the volume of lesions (Armstrong et al., 2016; Sharma et al., 2021). White matter is primarily composed of myelinated axons and various glial cells, which play an important role in signal transduction (Fields, 2008). Pathological changes in white matter mainly include local edema, demyelination, axon loss, oligodendrocyte loss, glial cell proliferation, and other histological changes (Hill et al., 2016). Damage to white matter also affects motor and sensory functions, resulting in long-term neurobehavioral syndrome and cognitive disorder (DeSouza et al., 2014; Filley and Kelly, 2018). Clinical studies have also shown that the degree of white matter damage in patients with neurological diseases is positively correlated with long-term motor and cognitive impairment (Hill et al., 2016). However, many previous animal model studies and clinical treatment experiments have largely ignored the treatment of white matter injury, and finally failed (Johnson et al., 2013). The most likely reason for this failure is that neuroprotective drugs are aimed at damage to only the neuron cell body but have no obvious protective effect on the part of white matter, which maintains connections between neurons, the cortex, and subcortical neurons. The treatment of white matter injury caused by secondary injury should also be given great attention. Therefore, research and development should be aimed at drugs for neurological diseases that protect not only the gray matter of the brain but also the white matter of the brain (Tabakman et al., 2004; Le Thuc et al., 2015; Takase et al., 2018).

## Synthesis and Metabolism of L-Serine

L-serine, a substance needed for cell proliferation, is synthesized by glial cells and is an important glial cell-derived neurotrophic factor, and its synthesis in the human body maintains nitrogen balance without food intake (Metcalf et al., 2018). L-serine synthesis and its derived biomolecules play an important role in the survival and differentiation of adjacent neurons and glial cells (Furuya and Watanabe, 2003; El-Hattab, 2016). At present, it is widely believed that serine is mainly synthesized in the body through the phosphorylation of 3-phosphoglycerate (3-PG), which is first isolated from the glycolysis pathway (Supplementary Figure 1). In the first stage of a series of enzymatic reactions, 3-PG is converted into phosphohydroxypyruvate (PHP) by 3-phosphoglycerate dehydrogenase (3-PGDH) and NADH, and PHP is then used to form phosphoserine through the activation of phosphoserine amino transferase (PSAT); finally, serine is formed through the dephosphorylation of phosphoserine via phosphoserine phosphatase (Locasale, 2013; Mattaini et al., 2016).

At the same time, L-serine is easily converted into glycine *in vivo*, which may prevent muscle degeneration and plays an important role in maintaining the balance of the CNS (Locasale, 2013). In contrast, serine is used to produce 5,10-methylenetetrahydrofolate, which is an important carbon and nitrogen donor in the synthesis of purines, pyrimidines and heme, thereby playing an important role in the growth and reproduction of organisms as well as the growth of normal blood cells (Verleysdonk and Hamprecht, 2000; Metcalf et al., 2018). Serine is also used to form sphingosine (nerve) and phosphatidylserine *in vivo*, which allows ceramide to be synthesized (de Koning et al., 2003). Ceramide is a neural sheath lipid composed of long-chain bases and sphingosine fatty acids that regulate immune function in animal and plant cell membranes (Castro et al., 2014). Serine is also used to form L-cysteine synthetic proteins to meet energy demands in animals (de Koning et al., 2003; Takagi and Ohtsu, 2017).

## L-Serine and D-Serine

A small part of the serine metabolized in the human body is transformed into D-serine by serine racemase under the activation of pyridoxal-5-phosphate as a coenzyme (Supplementary Figure 1). D-serine has been known as physiological functions, such as CNS development, and pathology, such as neuro-psychiatric and neurodegenerative diseases related to NMDA receptors dysfunction (Bardaweel et al., 2014). Substantial quantities of D-serine have been found to be present in the normal mammalian (mouse, rat, bull, and human) brain (Hashimoto et al., 1992; Nagata et al., 1995). It has been reported that D-serine levels and the ratio of D-serine to L-serine decrease in body fluid or brain in patients with schizophrenia, pediatric encephalopathy and AD comparable to that in controls (Yamamori et al., 2014; Soto et al., 2019; Le Douce et al., 2020). Le Douce et al. (2020) show that glycolysis is impaired in astrocytes in the early stages of disease in a mouse model of AD; this leads to the reduction of both L- and D-serine synthesis and to the alteration of synaptic plasticity and memory (Le Douce et al., 2020). However, Chronic D-serine supplementation has long been still under investigation. Slow and weak diffusion through the blood-brain barrier and potential nephrotoxicity have limited the clinical use of D-serine (Soto et al., 2019; Le Douce et al., 2020). Unlike D-serine, L-serine represents a more favorable therapeutic option because it is considered to be safe by the Food and Drug Administration (de Koning et al., 2003; Metcalf et al., 2018). L-serine has also been approved as a routine food additive, it is widely sold as a dietary supplement, and is well-tolerated, even at high doses (Table 1). Importantly, dietary supplementation with L-serine restores both deficits of L-serine and D-serine in the AD mouse model (Le Douce et al., 2020). These data raise the prospect of L-serine supplementation as a treatment option for neurological injury and diseases, and even for peripheral nervous systems (Table 1).

**TABLE 1:** Summary of the safe and effective doses of L-serine *in vivo* studies.

Research	Disease/injury model	Species	L-serine dose	Effects and mechanisms
Toxicology	Feeding toxicity study (Artom et al., 1945; Tada et al., 2010)	Rats	5.0% concentrations (p.o.) and 1.3 g/kg (p.o.)	No side effect
	ALS (Levine et al., 2017)	Human	30 g/day (p.o.)	No side effect
Brian injury	Focal cerebral ischemia (Wang et al., 2010a; Ren et al., 2013; Sun et al., 2014)	Rats	56, 168, or 504 mg/kg (i.p.)	Promoting endogenous NSC proliferation and microvascular proliferation (Sun et al., 2014)
				Mediating the small and intermediate-conductance Ca^2+^-activated K^+^ channels on the cerebral blood vessel endothelium (Ren et al., 2013)
				Neuroprotective effect may be mediated by activating glycine receptors (Wang et al., 2010a; Zhai et al., 2015)
	White matter demyelination (Wang et al., 2019) and traumatic brain injury (Zhai et al., 2015)	Mice	114, 342, or 1026 mg/kg (i.p.)	The therapeutic effect of L-serine may be related to its inhibition of the injury process, especially the inhibition of microglial activation and the release of inflammatory cytokines (Wang et al., 2019)
Neurodegenerative diseases	ALS patients (Levine et al., 2017)	Human	0.5, 2.5, 7.5, or 15 g/dose (p.o.)	L-serine appears to be generally safe for patients with ALS
	ALS/PDC (Cai et al., 2018)	Rats	62.5 mg/mL (i.p.) for 1 week	L-serine suppresses the erroneous incorporation of L-BMAA into proteins in the human nervous system
	Preclinical ALS/MND (Davis et al., 2020)	Vervet	210 mg/kg/day (p.o.)	L-serine reduces the amount of reactive gliosis and the number of protein inclusions in motor neurons
	HSAN1 (Garofalo et al., 2011)	Mice and Human	10% L-serine (w/w) (p.o.) and 10-week 200 or 400 mg/kg (p.o.)	L-serine supplementation also improved measures of motor and sensory performance as well as measures of male fertility
	Alzheimer’s disease (Le Douce et al., 2020)	Mice	10% L-serine for 2 months (p.o.)	L-serine supplementation as a treatment option for AD and possibly other neurodegenerative diseases
Peripheral neuropathy	Paclitaxel-induced painful peripheral neuropathy (Kiya et al., 2011)	Rats	0.01, 0.03, or 0.1 mmol/kg (i.p.)	L-serine derived from satellite cell that makes a decrease contributed to paclitaxel-induced painful peripheral neuropathy
Others	Oxidative stress and Sirt1/NF-κB signaling in the hypothalamus of aging mice (Zhou et al., 2018)	Mice	0.1, 0.2, or 0.5% (w/v) L-serine (p.o.)	Long-term L-serine administration reduces body weight by decreasing orexigenic peptide expression and reduces oxidative stress and inflammation during aging in mice by modulating the Sirt1/NF-κB pathway
	Human sleep (Ito et al., 2014)	Human	3 g L-serine for 4 days (p.o.)	Intaking L-serine before going to bed may improve human sleep

*ALS: amyotrophic lateral sclerosis; PDC: parkinsonism dementia complex model; MND: motor neuron disease; HSAN1: hereditary sensory and autonomic neuropathy type 1; p.o.: per os; i.p.: intraperitoneally injection.*

## Potential Mechanisms of L-Serine Against CNS Injury

**FIGURE 1:**
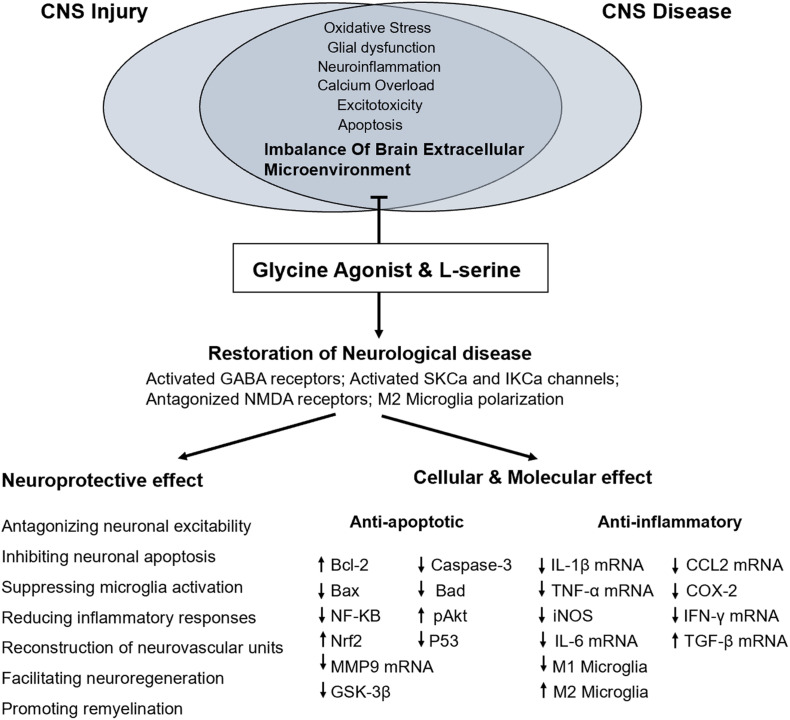
Proposed mechanism underlying the functional and molecular effects of L-serine treatment in CNS injury or diseases.

Central nervous system injury, such as that due to cerebral ischemia, anoxia, stroke, and TBI, is a common clinical pathological phenomenon that causes different degrees of damage to the brain. In the cascade following most hypoxic-ischemic brain injuries, neurons and glial cells release many excitatory amino acids, such as glutamate, while the reuptake of these excitatory amino acids is reduced (Fern and Matute, 2019; Pregnolato et al., 2019). Therefore, glutamate accumulates in the extracellular space, resulting in a large increase in the excitatory amino acid concentration (Fern and Matute, 2019). Activated excitatory amino acid receptors produce excitotoxicity, which is related to the mechanism of injury due to intracellular calcium overload, free radical proliferation, and an increase in nitric oxide production (Wang et al., 2007). L-serine, a neurotrophic factor, is an agonist of the glycine receptor, which is an inhibitory neurotransmitter receptor that hyperpolarizes neuron membranes when activated, thereby reducing the excitability of neurons (Chattipakorn and McMahon, 2002; Wang et al., 2010b). As an inhibitory neurotransmitter, L-serine promotes the proliferation of neural stem cells and has a neuroprotective effect in humans (Sun et al., 2014; Levine et al., 2017). In addition, L-serine, the main source of purine nucleotides and deoxythymidine monophosphate, plays an important role in cell proliferation (Verleysdonk and Hamprecht, 2000). Our previous study showed that L-serine had an outstanding therapeutic effect on lysophosphatidylcholine induced white matter injury in mice, which protected nerve cells and axons, promotion of oligodendrocyte proliferation, and remyelination (Wang et al., 2019). L-serine may have the ability to stimulate NSCs to differentiate into oligodendrocytes. Thus, L-serine may exert neuroprotective effects on the gray matter of the brain but also the white matter of the brain, and these effects are potentially mediated by multiple mechanisms (Figure 1).

## L-Serine Reduces Neuroexcitotoxicity

When the CNS is damaged due to conditions and injuries, such as ischemia, hypoxia, trauma, poisoning and other factors, excitatory neurotransmitters are released from nerve endings and abnormally accumulate in the synaptic space, producing excitatory toxicity; these processes result in overexcitation of adjacent neurons, sodium ion influx, cell degeneration, edema, and protein degradation, which causes irreversible cell necrosis and apoptosis in the CNS (Wang et al., 2007). Glutamate, the main excitatory neurotransmitter, is mainly involved in the transmission of synaptic signals and functions in learning and memory. In ischemic encephalopathy, neurons are over depolarized, and excitatory neurotransmitters (glutamate) activate many ionotropic receptors (Lau and Tymianski, 2010). Overactivation of the NMDA receptor promotes Ca^2+^ influx, causing the intracellular Ca^2+^ concentration to rise rapidly; therefore, excitatory neurotransmitters produce excitatory toxicity and cause the apoptosis and necrosis of neurons (Wang et al., 2007; Lau and Tymianski, 2010).

L-serine and its metabolites not only have significant effects on cell proliferation but are also necessary for specific brain functions (Metcalf et al., 2018). L-serine alleviates neurotoxicity by activating the glycine receptor, an important ion channel receptor in the CNS and peripheral system, and it has an important protective effect in the nervous system (de Koning et al., 2003; Sun et al., 2014). The glycine receptor is a chloride-selective transmembrane channel (Oda et al., 2007). When the glycine receptor is activated, the chloride channel opens, causing internal flow of chloride ions and neuron hyperpolarization (Lau and Tymianski, 2010). In addition, L-serine blocks the removal of Mg^2+^ from the NMDA receptor channel, which inhibits the opening of the NMDA receptor channel, thereby preventing the excitatory toxicity of glutamate and alleviating neuron depolarization and a series of reactions in a biological cascade (Ren et al., 2013; Sun et al., 2014). Therefore, glycine and other glycine agonists cause reactions involving Cl^–^ mediated by neurons in various brain regions, which prevent the excitotoxicity of neurons after injury and reduce the phenomena of apoptosis and necrosis in neurons. Our previous research confirmed that L-serine plays a neuroprotective role by activating glycine receptors in cerebral ischemia and in hippocampal neurons exposed to hypoxia or glutamate (Wang et al., 2010b; Ren et al., 2013; Sun et al., 2014). L-serine may also activate PI3K/Akt, which inhibits the activity of caspase-9, a protein hydrolase, to prevent the apoptosis cascade and reduce neuroexcitotoxicity (Luyendyk et al., 2008; Park et al., 2011).

## L-Serine Regulates Microglia Polarization

Microglia are immune cells in the central nervous system with a function similar to that of macrophages (Hu et al., 2015). After neurological disease, the activation of glial cells plays an important role in the inflammatory response (Wang et al., 2015; Karve et al., 2016). Microglial cells are the main participants in the inflammatory response, and various signals in the microenvironment of the injured area polarize macrophages/microglial cells in a time-dependent manner; that is, after macrophages/microglia are transiently polarized to a M2 phenotype, they are transformed into the more persistent M1 phenotype (Hu et al., 2015; Wang et al., 2015). M1 microglia secrete many proinflammatory factors, and reactive oxygen species cause cell death and the clustering of cell fragments, thereby increasing nerve cell death; however, M2 microglia secrete anti-inflammatory factors and phagocytize cell fragments to achieve nerve cell repair (Wang et al., 2013b; Hu et al., 2015). In the early stage of brain injury, microglia are in a state of overactivation, and more M1 microglia secrete many proinflammatory factors and increase nerve cell death, which is not conducive to the recovery of motor cognitive function after neurological disease (Wang et al., 2013b, 2015). Thus, if the number of M2 microglia greatly exceeds that of M1 microglia, neurotoxicity could be reduced.

Differentially expressed genes upon microglial inflammatory stimulation and L-serine treatment have been analyzed by whole-genome microarray, demonstrating that L-serine specifically increases peroxisome proliferator activated receptor γ (PPAR-γ) gene expression (Ding et al., 2020). PPAR-γ is an important transcription factor that regulates the polarization of macrophages and microglia (Song et al., 2017). After brain injury, the activation of PPAR-γ induces the polarization of M1 microglia toward a M2 phenotype, increasing the proportion of M2 microglia in the early stage of injury. Thus, inhibiting the release of inflammatory factors and increasing the effects of anti-inflammatory factors alleviate an excessive inflammatory response, saving damaged nerve cells, and reducing neurotoxicity. Inhibition of Akt also affects gene expression in M2 microglia (Martinez et al., 2008). Many neuroprotective drugs (neuroprotective agents, free-radical scavengers, and growth factors) promote the recovery of brain cognitive function by activating the Akt signaling pathway (Zhao et al., 2006; Wang et al., 2013a; Vergadi et al., 2017). L-serine may also activate the PI3K pathway, which is an essential step in the polarization of microglia toward a M2 phenotype in response to protein A or IL-4 (Wang et al., 2015, 2019). At the same time, L-serine may also activate PPAR-γ through the P13K/Akt/mTOR pathway to induce the polarization of microglia from the harmful M1 phenotype to the beneficial M2 phenotype and inhibit the cascade effect of inflammatory mediators (Wang et al., 2019; Ding et al., 2020), which increases the phagocytosis of cell fragments and inhibition of neurotoxicity, promoting recovery from neurological disease. In a model of white matter demyelination established through the *in vitro* co-culture of microglia and oligodendrocytes, we found that L-serine inhibits the secretion of inflammatory factors (TNF-α and IL-1β) to promote the repair of neurological function (Wang et al., 2019). It has also been confirmed that L-serine treatment promotes the proliferation of oligodendrocyte precursor cells at the organism level, indicating that L-serine reduces the degree of white matter damage in the brain by regulating the polarization of microglia after CNS damage (Wang et al., 2019).

## L-Serine Decreases Inflammation

Inflammation plays an important role in the pathological process of brain injury (Wang et al., 2013b). Although inflammation is the first line of defense against injury and infection, excessive inflammation aggravates brain tissue damage (Wang et al., 2015). The brain inflammatory response after CNS injury is mainly characterized by leukocyte recruitment, glial cell activation and an increase in cytokines and inflammatory factors (Dokalis and Prinz, 2019). The cascade effect of these inflammatory mediators leads to the aggravation of inflammation, which is not conducive to the recovery of neural function (Simon et al., 2017). Moreover, infiltrated neutrophils release proteases and oxidases, which aggravate secondary brain injury (Corrigan et al., 2016). Glial cells are mainly involved in regulating inflammation when the central nervous system is damaged (Karve et al., 2016). TNF-α, IL-6, IL-1β, and other proinflammatory factors are all expressed at high levels (Karve et al., 2016; Long et al., 2017). The TNF-α, IL-6, and IL-1β proinflammatory cytokines play an important role in regulating the inflammatory cascade, in which IL-6 activates microglial cells and increases inflammation in the brain, which is not conducive to the recovery of neural function (Raivich et al., 1999; Karve et al., 2016).

A comparison of mRNA expression profiles has shown that TBI upregulates nine mRNAs (Cxcl10, IL-18, IL-16, Cd70, Mif, Ppbp, Ltb, Tnfrsf11b, and Faslg) in the blood to drive proinflammatory and/or neurodegenerative processes (Chio et al., 2017). TBI also upregulates 13 mRNAs (Ccl19, Ccl13, Cxcl19, IL-10, IL-22, Bmp6, Ccl22, IL-1rn, Ccl2, IL-7, Bmp7, Gpi, and Ccl17) in the blood to drive anti-inflammatory and/or neurodegenerative events (Zhai et al., 2015; Chio et al., 2017). Our research has confirmed that L-serine inhibits the proliferation and activation of microglia and astrocytes but also reduces the secretion of TNF-α, IL-6, IL-1β, and other proinflammatory factors to reduce inflammation and improve neurological function disorder (Sun et al., 2014; Zhai et al., 2015). When glycine receptor inhibitors are applied, L-serine inhibits the release of inflammatory factors, and the activation of glial cells is significantly inhibited (Zhai et al., 2015). This finding shows that L-serine reduces the secretion of proinflammatory cytokines by activating the glycine receptor to reduce the inflammatory response of the brain and plays a protective role in neurological function after brain injury.

## L-Serine Improves Cerebral Blood Flow (CBF)

The brain regulates changes in vascular pressure, and CBF is improved through the vasoactive response of the cerebral arteries and arterioles. This myogenic tension significantly improves the resistance of cerebral vessels, including the MCA, pial arteries, and parenchymal arterioles (Ren et al., 2013; Palomares and Cipolla, 2014). Some pathological phenomena after CNS injury, such as arteriosclerosis and cerebral thrombosis, often lead to serious hypoxic-ischemic brain injury and a decrease in CBF, leading to tissue necrosis and brain dysfunction (Wang et al., 2011, 2013a). In general, cerebral endothelial cells regulate vascular tension by producing vasoactive factors, nitric oxide (NO), prostacyclin, and endogenous hyperpolarizing factor (EDHF; Yada et al., 2003). Both prostacyclin and NO are endothelium-derived vasodilators that mainly play a role in vasodilation of the large arteries, while EDHF plays a role in the vasodilation of arteries less than 500 μm in diameter, especially those less than 100 μm in diameter (Rath et al., 2009). The interaction of EDHF with endothelial cells increases the release of Ca^2+^ in the endoplasmic reticulum and the Ca^2+^ concentration in the cytoplasm (Ferreiro et al., 2004). Moreover, EDHF also activates the small conductance calcium-dependent potassium channel (SKCa) and the intermediate conductance calcium-dependent potassium channel (IKCa; Ren et al., 2013). The SKCa and IKCa channels are found in the middle cerebral artery (McNeish et al., 2006). Activation of these two channels causes expansion of the middle cerebral artery. At rest, blockade of these two channels reduces CBF by approximately 15%, while activation of these two channels increases CBF by approximately 40% (Cipolla et al., 2009; Behringer, 2017). The activation of these two channels also causes the outflow of intracellular potassium ions and the hyperpolarization of endothelial cells, and due to the connection between smooth muscle cells and endothelial cells, the activation of SKCa and IKCa channels also causes the hyperpolarization of smooth muscle cells, promoting the vasodilation of arteries and improving CBF (McNeish et al., 2006; Cipolla et al., 2009; Behringer, 2017).

It has also been confirmed that L-serine plays a role in vasodilation after focal cerebral ischemia injury, unlike EDHF (Mishra et al., 2008; Ren et al., 2013). L-serine can be used as an agonist of SKCa and IKCa channels in vascular endothelial cells, changing the membrane potential and calcium homeostasis in endothelial cells as well as hyperpolarizing endothelial cells and increasing the concentration of calcium ions in the cytoplasm to regulate cerebrovascular resistance, relax vascular pressure and increase CBF in the ischemic area (Ren et al., 2013; Zhai et al., 2015). Therefore, L-serine also has a neuroprotective effect by improving CBF after cerebral ischemia.

## L-Serine Promotes the Survival, Proliferation and Differentiation of Neural Stem Cells (NSCs)

Many *in vitro* experiments have confirmed that L-serine promotes the proliferation and differentiation of NSCs, which may be related to the protective role of L-serine-mediated activation of the PI3K/Akt/mTOR pathway (de Koning et al., 2003; Sun et al., 2014; Metcalf et al., 2018). In contrast, L-serine also inhibits the activation of caspase-3, which is related to the mechanism by which L-serine inhibits NSC apoptosis (Zhai et al., 2015). When cells are damaged, the apoptotic factor, cytochrome c, is released from damaged mitochondria (Gonzalez-Arzola et al., 2019). Cytochrome c forms apoptotic bodies in the cytoplasm with apoptotic protease activating factor (Apf-1) and procaspase-9, which activate caspase-3 and caspase-9, leading to cell apoptosis (Donovan and Cotter, 2002). In addition, the extracellular Fas-binding death receptor activates caspase-8 or -10 in the exogenous apoptotic pathway, which then directly activates caspase-3 and induces the release of cytochrome c (Mattson, 2000; Ning et al., 2004). Hence, L-serine may inhibit NSC apoptosis and promote the proliferation and differentiation of NSCs by activating the Akt signaling pathway and inhibiting caspase-3 activation.

White matter refers to areas of the CNS that are mainly made up of myelinated axons, and oligodendrocytes are myelinating cells (Barateiro et al., 2016). Oligodendrocytes mainly arise from oligodendrocyte precursor cells (OPCs), but some oligodendrocytes arise from NSCs (Harauz et al., 2009; Gregath and Lu, 2018). Activation of Akt by L-serine promotes the proliferation and survival of NSCs after brain injury, which plays an important role in myelin recovery (Zhang et al., 2010; Wang et al., 2013a; Tian et al., 2019). Moreover, L-serine is an endogenous substance in the body, and it stimulates the receptors for the inhibitory amino acid glycine and GABA receptors (Wang et al., 2010b). Previous studies have shown that some neurotransmitters have an activating effect on endogenous NSCs, such as activating the inhibitory neurotransmitter GABA receptor and finally promoting the proliferation of NSCs (Ge et al., 2006). The GABA receptor, a neurotransmitter, activates endogenous NSCs, and promotes NSC proliferation (Ge et al., 2006). Altogether, L-serine may also stimulate NSCs to differentiate into oligodendrocytes.

## Summary and Prospects

Many experiments have demonstrated that long-term treatment with L-serine increases the levels of neurotrophic factors in the tissue of the injured side of the brain, promoting the proliferation of endogenous NSCs and the repair of neurological function. L-serine regulates microglial polarization, promoting the repair of white matter damage and reducing inflammation. L-serine also reduces neurotoxicity and inhibits the release of inflammatory factors by activating the glycine receptor. In an ischemic brain injury model, L-serine has been found to activate potassium and calcium channels on endothelial cells to increase cerebral blood flow in the ischemic area. As an endogenous amino acid, the safety of L-serine has also been experimentally tested in a phase I human clinical trial, demonstrating that L-Serine repairs neurological function after secondary injury. The neuroprotective effects of L-serine described herein are so compelling it is tempting to consider how they might translate to humans and obtain satisfactory effects for the clinical use of L-serine. Further studies are also necessary to determine whether, where, and when the levels of L-serine change during disease progression.

## Author Contributions

GW and ZJ conceived, organized, and supervised the work. LY and YS contributed to manuscript writing and literature search. GW prepared and revised the manuscript. All authors contributed to the article and approved the submitted version.

## Conflict of Interest

The authors declare that the research was conducted in the absence of any commercial or financial relationships that could be construed as a potential conflict of interest.

## Publisher’s Note

All claims expressed in this article are solely those of the authors and do not necessarily represent those of their affiliated organizations, or those of the publisher, the editors and the reviewers. Any product that may be evaluated in this article, or claim that may be made by its manufacturer, is not guaranteed or endorsed by the publisher.

## Supplementary Material

Supplementary Figure 1 Pathway of L-serine synthesis and metabolism. L-serine originates from an intermediate of the glycolysis pathway, and is mainly synthesized in the astrocyte body through the phosphorylation of 3-phosphoglycerate (3-PG). In the first stage of a series of enzymatic reactions, 3-PG is converted into phosphohydroxypyruvate (PHP) by 3-phosphoglycerate dehydrogenase (3-PGDH) and NADH, and PHP is then used to form phosphoserine through the activation of phosphoserine amino transferase (PSAT); finally, serine is formed through the dephosphorylation of phosphoserine via phosphoserine phosphatase. 3-PGDH, and a small neutral amino acid transporter (ASCT1) are preferentially expressed in adult astrocytes. A small part of the serine metabolized in the human body is transformed into D-serine by serine racemase under the activation of pyridoxal-5-phosphate as a coenzyme. *In vivo*, L-serine is also used to produce or form glycine, 5,10-methylenetetrahydrofolate, sphingosine and phosphatidylserine, etc. Notably, sphingolipids and phosphatidylserine are indispensable components of the cell membrane that play an important role in the development of CNS function.
