# Supplementary figures and images for: L-Serine, an Endogenous Amino Acid, Is a Potential Neuroprotective Agent for Neurological Disease and Injury
Source: Front Mol Neurosci. 2021 Sep 6;14:726665. doi: 10.3389/fnmol.2021.726665 (PMC8450333; doi:10.3389/fnmol.2021.726665)

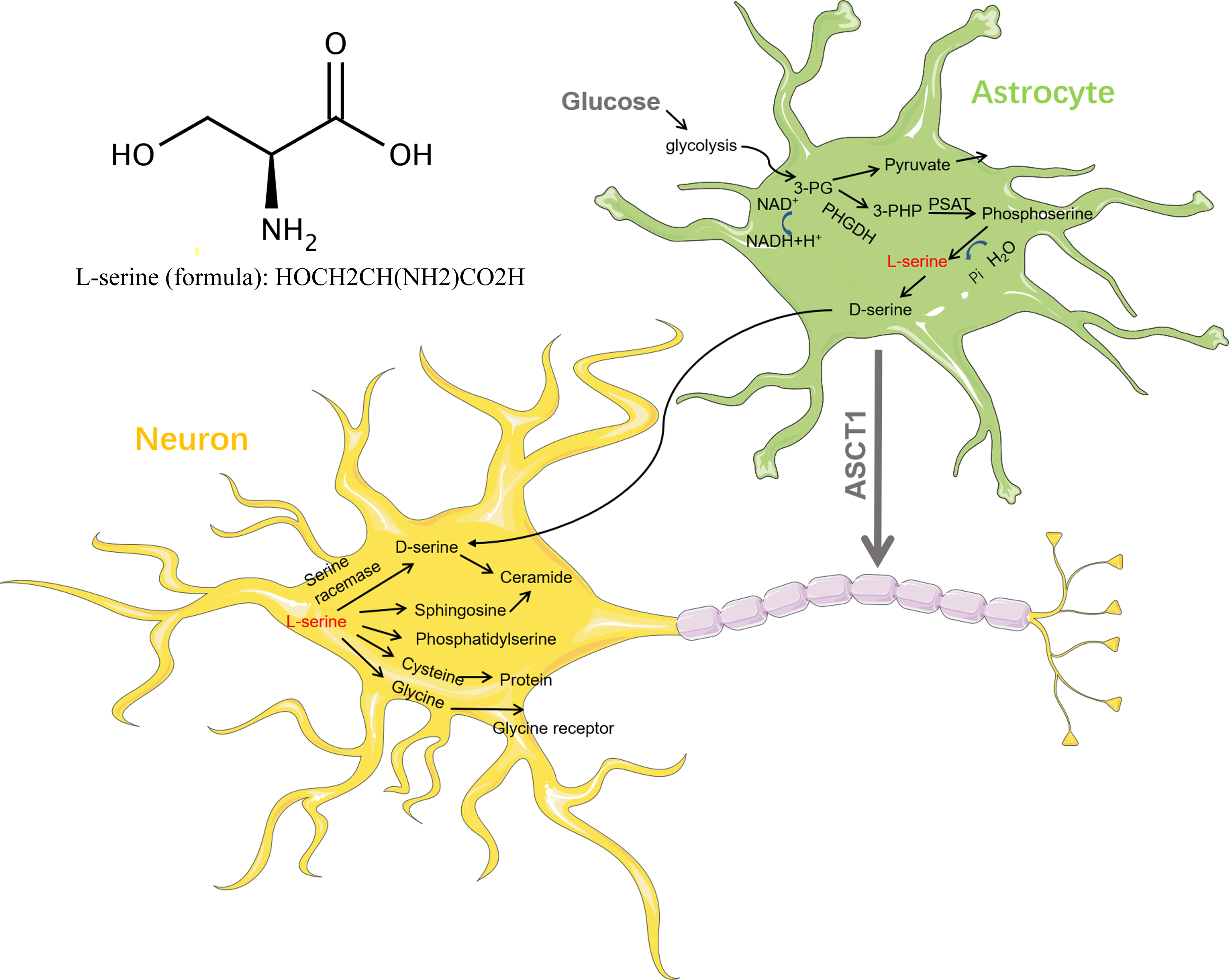

Supplement: Supplementary Figure 1 — Pathway of L-serine synthesis and metabolism. L-serine originates from an intermediate of the glycolysis pathway, and is mainly synthesized in the astrocyte body through the phosphorylation of 3-phosphoglycerate (3-PG). In the first stage of a series of enzymatic reactions, 3-PG is converted into phosphohydroxypyruvate (PHP) by 3-phosphoglycerate dehydrogenase (3-PGDH) and NADH, and PHP is then used to form phosphoserine through the activation of phosphoserine amino transferase (PSAT); finally, serine is formed through the dephosphorylation of phosphoserine via phosphoserine phosphatase. 3-PGDH, and a small neutral amino acid transporter (ASCT1) are preferentially expressed in adult astrocytes. A small part of the serine metabolized in the human body is transformed into D-serine by serine racemase under the activation of pyridoxal-5-phosphate as a coenzyme. In vivo, L-serine is also used to produce or form glycine, 5,10-methylenetetrahydrofolate, sphingosine and phosphatidylserine, etc. Notably, sphingolipids and phosphatidylserine are indispensable components of the cell membrane that play an important role in the development of CNS function. [file Image_1.TIF]
